# The impact of sediment, fresh and marine water on the concentration of chemical elements in water of the ice-covered lagoon

**DOI:** 10.1007/s11356-021-14936-w

**Published:** 2021-06-25

**Authors:** Magdalena Bełdowska, Agnieszka Jędruch, Dorota Sieńska, Wojciech Chwiałkowski, Artur Magnuszewski, Ryszard Kornijów

**Affiliations:** 1 Institute of Oceanography, University of Gdańsk, Piłsudskiego 46, 81-378 Gdynia, Poland; 2 Advanced Environmental Analysis Laboratory, Elbląg Technology Park, Sulimy 1, 82-300 Elbląg, Poland; 3 Department of Hydrology, University of Warsaw, Krakowskie Przedmieście 30, 00-927 Warsaw, Poland; 4 Department of Fisheries Oceanography and Marine Ecology, National Marine Fisheries Research Institute, Kołłątaja 1, 81-332 Gdynia, Poland

**Keywords:** Remobilisation, Inflow, Metals, Vistula Lagoon, Estuary, Icing

## Abstract

The common use of chemical elements by man has been contributing to their extraction for centuries. As a consequence, they have been directly or indirectly introduced into the biogeochemical cycle. In the framework of many conventions, mining and processing of elements are currently subject to many restrictions. However, their large load that has already been deposited in the soil and bottom sediments can be remobilised and enter the food chain. The identification of factors favouring this process is very important, especially during the period of adopting new legal regulations on limiting the emission of pollutants. It became possible in February 2018 during the persistence of ice cover on the lagoon’s surface. This allowed observation of processes, the effect of which in the absence of ice is blurred by wind mixing water. Therefore, an investigation of sources of 25 elements in a lagoon of the southern Baltic has been undertaken, based on the example of the Vistula Lagoon. The results point to the remobilisation of chemical elements (including the toxic ones) from land and bottom sediments, where they have been deposited for decades. These processes led to the accumulation of metals in certain areas of the lagoon. It may result in their uptake and accumulation in the benthic organisms inhabiting the lagoon and further transfer in the food chain. It is of major importance as the lagoons in the southern Baltic fulfil many essential functions in the scope of tourism, economy, and fishery. Thanks to restrictions on the quality of wastewater and the emission of pollutants, it has been noticed a substantial “purifying” effect of rivers, too.

**Supplementary Information:**

The online version contains supplementary material available at 10.1007/s11356-021-14936-w.

## Introduction

**Fig. 1:**
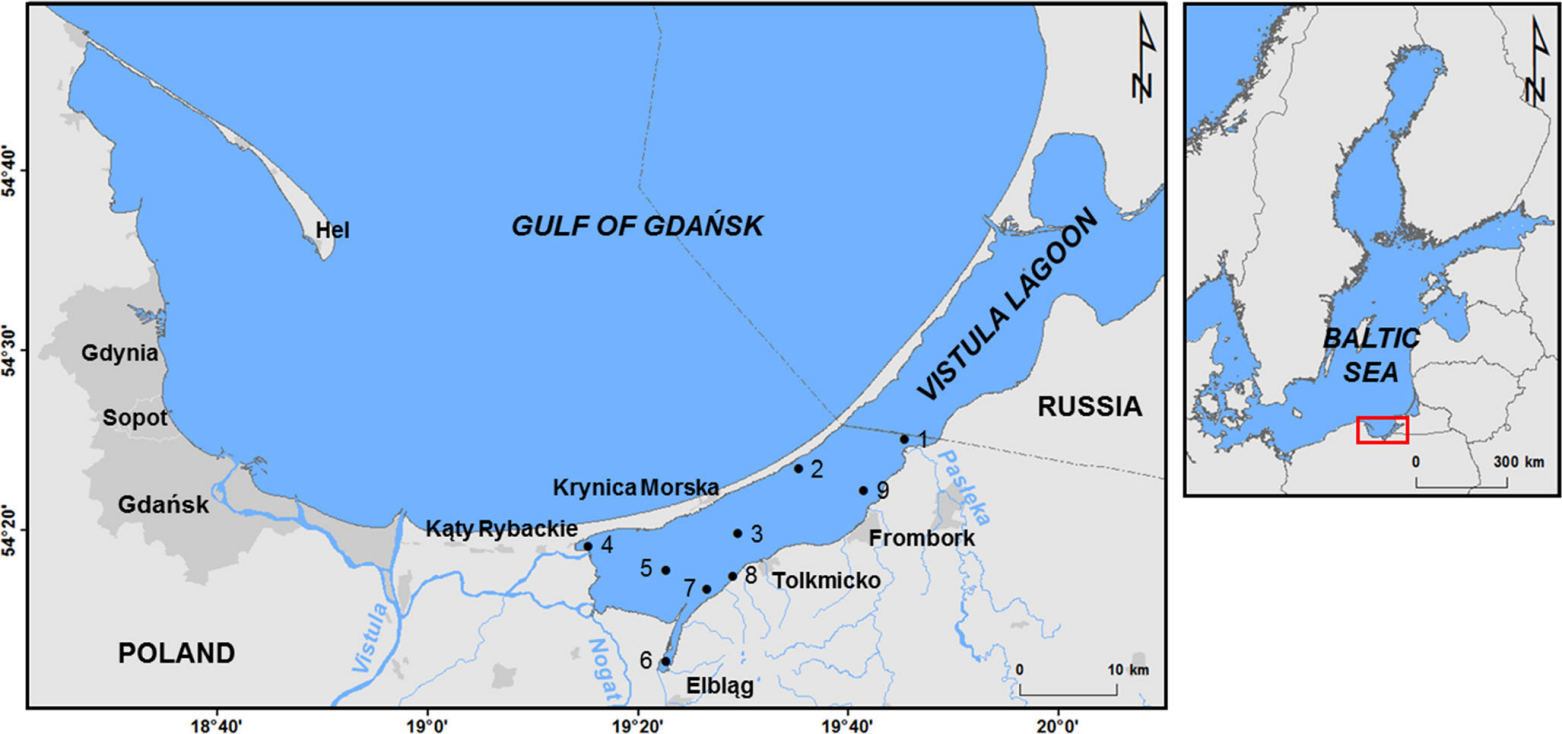
Location of sampling station in the Polish part of the Vistula Lagoon (southeastern Baltic Sea)

Intensive industrial development has contributed to the extraction of natural elements from the Earth. The mining itself together with the processing, production, and then disposal of used products have been contributing to the introduction of chemical elements to the cycle and food web (Helios-Rybicka 1996). Elements emitted and released to the environment can be transported over long distances via the atmosphere and then be deposited on land and water surfaces, often far from their source (Petersen 1999). After being deposited on land, the elements may be stored in soils or reach the aquatic environment along with surface run-off (Bełdowska et al. 2016). As a result, they can accumulate in the aquatic organisms and often biomagnify across the successive trophic levels (Sokołowski 2009; Roldán-Wong et al. 2018). Some elements are toxic and have no positive role in living organisms (e.g. cadmium, lead). Part of them is needed in small quantities but strongly toxic when the threshold is exceeded (e.g. arsenic, selenium). Others are necessary as macroelements, but also dangerous in high doses (e.g. zinc, copper). Their common presence in the natural environment can be hazardous to the health or even life of living organisms, including humans (Kabata-Pendias and Mukherjee 2007). Due to the increasing awareness on the toxicity of chemical substances, restrictions aimed at reducing their emission and reemission have been implemented for the last few decades (HELCOM 2018). Nonetheless, a vast load of pollutants is already deposited in the soil and marine sediments from where it can be released to the environment and introduced to the food chain (Pempkowiak et al. 1999; Szefer 2002; Jędruch et al. 2019). A number of tasks have been recently implemented in many countries, aimed at minimising anthropogenic threats, particularly in strongly polluted areas. Rivers serve as transport routes of chemical elements in the environment. They transport products of rock weathering and soil erosion with flowing water. River water transports suspended particles and solutions, both of which can contain metals. Part of the pollution load becomes immobilised in river sediments, although the final place of deposition of metals is in the marine environment, especially in waters of estuaries and coastal lagoons. Therefore, natural processes such as outflow of chemical elements from land (particularly due to intensive precipitation or coastal erosion) or resuspension from bottom sediments are currently gaining increasing importance (Bełdowska et al. 2016). This way, the chemical elements can be transported with surface run-off from land to the coastal zone of seas. In water bodies, they can be transported with bottom currents from polluted areas to clean ones (Rennera et al. 1998; Szefer 2002; Krek et al. 2019). The discussed processes are particularly important in estuaries and lagoons, where water exchange with the open sea is strongly limited. The Vistula Lagoon, located in the south-eastern part of the Baltic Sea, is an example of such a water body (Fig. 1).

Owning to high geological diversity, Poland is a country with large mineral resources and a rich tradition of mining. These are in particular traditional raw materials such as hard coal or lignite. The ores of copper, zinc, lead, iron or sulfur and the rare earth elements (REE) deposits are also located on its territory. However, the most valuable deposits are situated in the south of the country, while the lands of northern Poland have no significant mineral deposits (PIG 2017). Therefore, they do not directly affect the concentration level of the elements in the waters of the Vistula Lagoon. As a result of the presence of lock gate on the Nogat and Szkarpawa Rivers, the inflow of water from the Vistula River, the largest river flowing into the Baltic Sea in the area (Fig. 1), is very limited. As a consequence, the input of pollutants transported with the waters of the Vistula River, primarily deposited in the Gulf of Gdańsk, is also inhibited (Szefer 2002; Szefer and Grembacka 2009; Saniewska et al. 2014; Jędruch et al. 2015). This is confirmed among others by results of research on heavy metals in surface sediments or macroalgae — both their concentrations and enrichment factor (EF) measured in the area of the Vistula Lagoon were lower than in the Gulf of Gdańsk (Haroon et al. 1995; Glasby and Szefer 1998; Sokołowski 2009; Szefer et al. 2009; Bełdowska and Sokołowski 2018). Concentrations of trace metals in the Vistula Lagoon also show spatial variability. Higher than average concentrations of metals such as cadmium, lead or silver have been recorded among others in the area neighbouring the harbour in Elbląg (Szefer and Grembacka 2009). However, the identification of other sources of toxic or potentially toxic elements in the lagoon is relatively difficult due to many co-occurring factors. One of the possibilities of more detailed identification of the sources of chemical substances is the appearance of ice cover, permitting observing processes the effect of which is blurred by the wind mixing in the ice-free season.

The study aimed to investigate the sources and distribution pattern of chemical elements (sodium Na; potassium K; magnesium Mg; strontium Sr; calcium Ca; aluminium Al, antimony Sb; arsenic As; chromium Cr; copper Cu; iron Fe; lead Pb; manganese Mn; molybdenum Mo; nickel Ni; selenium Se; silver Ag; uranium U; vanadium V; zinc Zn; cadmium Cd; cobalt Co; thallium Tl; silicon Si) as well as bromides (Br^-^), nitrates (NO_3_^-^), chlorides (Cl^-^), and sulphates (SO_4_^2-^) in a southern Baltic lagoon based on the example of the Vistula Lagoon under ice conditions.

## Materials and methods

### Sampling and field measurements

The research has been conducted in the Vistula Lagoon (Fig. 1). Over its almost entire length, the lagoon is separated from the Gulf of Gdańsk with the Vistula Spit and is connected with the open sea through the Strait of Baltiysk with a width of only 380 m. It is through the strait that water exchange with the Baltic Sea occurs, whereas the direction of the exchange depends on hydrological-meteorological conditions, such as water level difference or wind direction. The inflow of marine waters largely determines the water balance of the Vistula Lagoon and constitutes approximately 80% of the water flowing into the lagoon. River water supply accounts for 17%, and precipitation and groundwater discharge are low (Matciak and Chyła 2018). The largest rivers flowing into the Vistula Lagoon from the territory of Poland show the following average run-off values (period 1998–2000): Nogat and Szkarpawa 0.763 km^3^ a^-1^, Pasłęka 0.657 km^3^ a^-1^, Elbląg 0.231 km^3^ a^-1^, and Bauda 0.086 km^3^ a^-1^. Rivers flowing into the Vistula Lagoon from the territory of Russia and their average run-off are as follows: Pregolya 1.81 km^3^ a^-1^, Prokhladnaya 0.272 km^3^ a^-1^, Mamonovka 0.162 km^3^ a^-1^, and Nelma 0.065 km^3^ a^-1^ (Witek et al. 2010).

Research on ice phenomena on the Vistula Lagoon is conducted based on observations on hydrological stations of the Polish Institute of Meteorology and Water Management (Łazarenko and Majewski 1975). On the Vistula Lagoon, ice cover develops on average in early December, the latest at the beginning of the third decade of January. Ice in the form of shuga (new ice composed of spongy, white lumps a few cm across) or landfast (ice that is "fastened" to the coastline or to the sea floor along shoals) first develops in coastal areas in bays, harbour basins, and on average 3–4 days later in the open part of the lagoon. The southern shore cools faster, and water inflow from the bay through the Strait of Baltiysk inhibits ice development. Ice cover may disappear and form again during warmer winters. Ice melting usually begins in late February or early March. Ice disintegration usually begins in the vicinity of the Strait of Baltiysk penetrated by warm marine waters, and then in river mouths. Wind plays an important role, considerably accelerating the process of melting of the ice cover (Herman 2018).

The development of ice cover in the lagoon was monitored based on the analysis of Sentinel-1 SAR satellite images, with a temporal resolution of 2–3 days, as described by Kornijów et al. (2020). The thickness of the ice cover over sampling points represented the solid ice phase. Other forms of ice were not described due to problems with their detection by visual interpretation of the satellite images. Moreover, the study employed information included in the Polish Ice Reports (IMGW 2018) and obtained from the administration of the harbour in Tolkmicko (Fig. 1). The ice season 2017/2018 in the Vistula Lagoon lasted for 96 days.

The research was carried out on 23 February 2018, in the second episode of ice formation on the Vistula Lagoon. The thickness of the ice cover was approximately 10 cm, and the ice was covered by a 2–5-cm layer of snow. Water was sampled from under ice from nine stations in the Polish zone of the lagoon (Fig. 1). At each site, the samples were collected after making a hole in the ice (using a hand ice auger) approximately 40 cm in diameter. Water samples from shallow sites (up to a depth of 2 m) were collected from the surface to the bottom using tube samplers with a diameter of 4 cm and length of 120–200 cm, closed at the top with a plug. Water was collected every several tens of centimetres until filling an 11-L container. A 0.5-L water subsample was taken from the total volume for the physico-chemical analyses. At three deepest sites (3, 5, 9), water samples were collected separately from three layers: at the surface, from the middle of the water column (using a 5-L Niskin water sampler), and at the bottom (using a horizontal version of Niskin sampler).

Water samples were filtered in a clean laboratory through membrane filters made of PTFE with a pore diameter of 0.45 μm, acidified with concentrated (65%) nitric acid (V) with purity for trace metal analysis until a pH of approximately 1. They were stored at a temperature of 5 °C until the analysis. Water samples for the analysis of ion concentration were not acidified. They were stored at a temperature of −20 °C until the analysis.

Measurement of pH and electrolytic conductivity was performed using a multifunction measurement device ELMETRON CX-701 with the application of a pH probe by Ionode and a conductometric sensor EC-60 by Elmetron. Water transparency was measured with a Secchi disc (SD), and salinity, temperature, and oxygen concentration in water with a portable Elmetron CPC-401 meter with appropriate probes.

### Chemical analysis

Concentrations of chemical elements (Na, K, Mg, Sr, Ca, Al, Sb, As, Cr, Cu, Fe, Pb, Mn, Mo, Ni, Se, Ag, U, V, Zn, Cd, Co, Tl) were determined by means of an inductively coupled plasma–mass spectrometer ICP-MS (Agilent Technologies 7700x). The analyses involved a multi-element model (LGC) with a matrix of 5% solution nitric acid (V). Concentrations of sodium, potassium, calcium, and magnesium ions and also bromides, nitrates, chlorides, and sulphates were analysed using an ion chromatograph Thermo Scientific Dionex ICS 1100. The method was validated for each determined element (ICP-MS) by using tuning solution (Multi Element Aqueous CRM Tuning Solution for Agilent 7500cs Matrix: 2% HNO3) and calibration solution (Multi Element Aqueous CRM Environmental Calibration Standard Blend A, Matrix 5% HNO3/tr. Tartaric Acid/tr. HF), a solution to check the calibration (Multi Element Aqueous CRM Environmental ICV Standard A Matrix: 5% HNO3/tr. Tartaric Acid/tr. HF) as well as certified reference material (Reference Material for Measurement of Elements in Surface Waters, SPS-SW2 Batch134). The limit of quantification and detection was determined for each element, as well as precision and accuracy (Table S1). Silicon concentration in water was determined using a cuvette test (method 8185 Hach Lange) with the use of a spectrophotometer UV-VIS HACH LANGE DR 2800. The analysis of total organic carbon (TOC) concentration was performed by means of an analyser TOC-LCPH/CPN SHIMADZU. Certified reference material PERADE-09 was used as reference material.

### Processing of results

A map of the study area was prepared in ArcMap 10.4.1 software (ESRI) with the WGS1984 geographic coordinate system and the UTM zone 34N for data projection. Part of the basic spatial data was provided courtesy of the GIS Centre, University of Gdańsk (www.ocean.ug.edu.pl/~oceju/CentrumGIS). The access to the satellite data from the Sentinel-1 satellite was provided by the European Space Agency (ESA) via the Copernicus Platform (www.scihub.copernicus.eu). Sentinel-1 Interferometric Wide (IW) Swath Mode data were geometrically corrected and registered to UTM coordinates as Ground Range Detected Geo-referenced Product (GRD). Data were obtained from the European system of Earth observation Copernicus at address https://scihub.esa.int/. For the processing of Sentinel-1 images, the SNAP free software was used. Subsections of the original scenes have been extracted covering the Vistula Lagoon area. After geometric correction, it has been obtained two channels with different polarisation: Amplitude_VH and Amplitude_VV. To improve the interpretation colour composition, RGB has been created using the following channels: Red — Aplitude_VH, Green — Amplitude_VV, Blue — log10 (Amplitude_VV). The location of the sampling points was displayed on the top of the images and visual interpretation used to find to recognise the type of ice cover or open water surface at a given point. Sentinel-1 SAR images are showing the pixel brightness which is proportional to backscatter, better reflection of the electromagnetic radiation means brighter pixel recorded in amplitude channels. Radar signals do not penetrate the ice so we have only information on the character of the ice or water surface. The spatial distribution of concentrations of the investigated chemical elements and other environmental parameters in the Vistula Lagoon was estimated via the inverse-distance weighting (IDW) interpolation method (Burrough et al. 2015).

## Results

### Ice phenomena

**Fig. 2:**
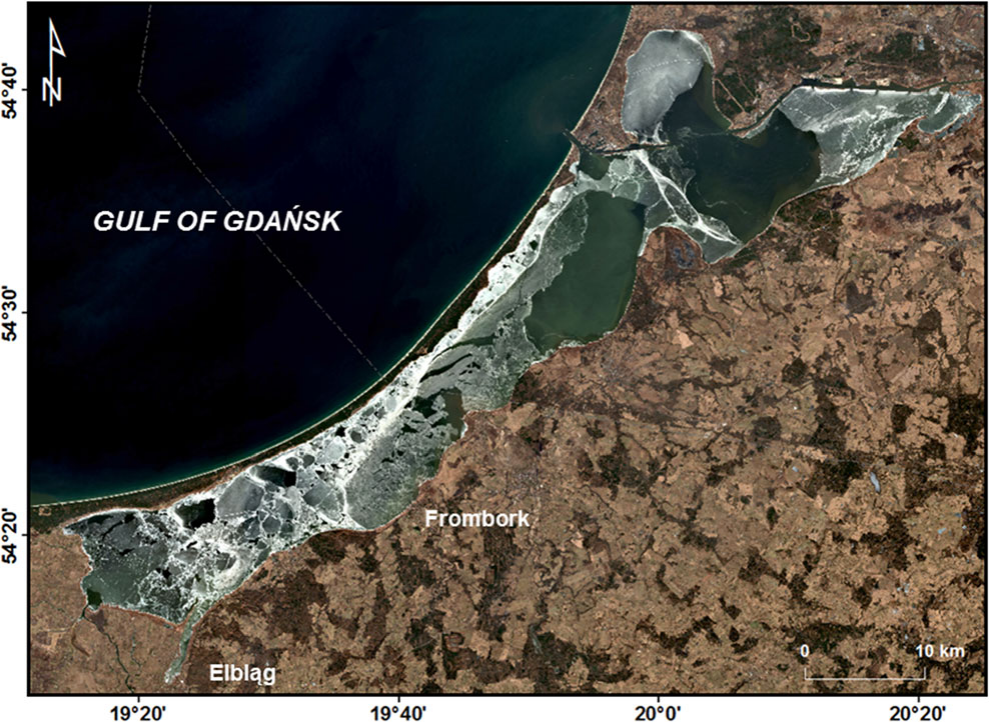
The extent of the impact of riverine water on the ice cover of the Vistula Lagoon recorded on a satellite image from Sentinel-1 radar backscatter bands of polarisation (VV), recorded on 31st January 2019

The analysis of Sentinel-1 satellite images showed that the first episode of ice cover on the Vistula Lagoon was short, and lasted from 14 to 29 January 2018. In the second episode, ice appeared on 7 February and persisted until 26 March 2018. The maximum duration of permanent ice cover over measurement points was 61 days (Kornijów et al. 2020). The thickness of the ice cover averaged 10 cm, in some areas reaching even 25 cm (IMGW 2018). The duration of ice cover over sampling sites is shown in Fig. S1. The results show a shorter duration of ice cover on stations 1, 4, and 6. Station 1 was affected by inflows of warm and salty water from the Strait of Baltiysk. Station 4 is under the influence of the Wisła Królewiecka and Szkarpawa Rivers, while station 6 is located in the vicinity of the mouth of the Elbląg River (Figs. 1 and 2).

### Chemical elements in water under ice cover

The highest concentration in the water of the Vistula Lagoon on nine stations under the ice was reached by ions and macroelements (Table 1). They were dominated by chlorides (mean: 838 mg L^-1^), although their concentration varied in the broadest range among the chemical elements, from 35 to 1778 mg L^-1^ (relative standard deviation RSD: 60%). Another ion showing a high concentration was sodium, with a mean concentration more than twice lower than that of chlorides (mean: 364 mg L^-1^), although it showed an approximate relative variability in the waters of the lagoon (RSD: 58%). Concentrations of the remaining ions and macroelements were as follows: sulphates (mean: 158 mg L^-1^) > calcium (mean: 70 mg L^-1^) > magnesium (mean: 44 mg dm L^-1^) > potassium (mean: 17 mg L^-1^) > nitrates (mean: 8 mg L^-1^) > silicon (mean: 6 mg L^-1^) > bromides (mean: 3 mg L^-1^). The elements showed variability in a range from 20% for Ca to 47% for Br^-^.

**Table 1:** Concentration of the chemical elements and ions in waters of the Vistula Lagoon

Chemical element/ion	Unit	Mean	SD	Min	Max	RSD (%)
Na	mg L^-1^	364.36	211.87	24.00	753.06	58
K	mg L^-1^	16.75	7.13	5.40	29.95	43
Mg	mg L^-1^	43.49	13.91	11.00	83.77	32
Sr	μg L^-1^	503.40	117.53	294.77	723.50	23
Ca	mg L^-1^	69.55	13.89	55.70	98.06	20
Al	μg L^-1^	1.10	0.70	0.31	2.63	64
Sb	μg L^-1^	0.24	0.08	0.14	0.41	33
As	μg L^-1^	0.74	0.16	0.46	1.01	22
Cr	μg L^-1^	1.67	0.75	0.63	3.62	45
Cu	μg L^-1^	6.01	2.60	1.94	11.21	43
Fe	μg L^-1^	7.84	6.49	2.69	27.54	83
Pb	μg L^-1^	2.06	6.25	0.04	25.41	303
Mn	μg L^-1^	76.38	78.52	2.50	277.48	103
Mo	μg L^-1^	0.85	0.14	0.61	1.06	16
Ni	μg L^-1^	2.32	0.87	1.15	4.60	38
Se	μg L^-1^	1.71	0.74	0.15	3.05	43
Ag	μg L^-1^	0.05	0.04	0.01	0.18	80
U	μg L^-1^	1.15	0.36	0.74	2.09	31
V	μg L^-1^	0.38	0.06	0.27	0.50	16
Zn	μg L^-1^	5.40	3.45	2.30	15.78	64
Cd	μg L^-1^	0.05	0.05	0.01	0.23	100
Co	μg L^-1^	0.26	0.12	0.12	0.53	46
Tl	μg L^-1^	0.01	0.00	0.00	0.01	0
Si	mg L^-1^	5.53	1.06	3.40	7.20	19
Br^-^	mg L^-1^	2.59	1.21	0.88	4.74	47
NO_3_^-^	mg L^-1^	7.91	3.25	0.74	0.74	41
Cl^-^	mg L^-1^	838.37	506.25	35.00	1777.82	60
SO_4_^2-^	mg L^-1^	158.08	60.34	59.00	269.41	38

In the case of trace metals, decidedly highest concentrations were analysed for Sr (mean: 503 μg L^-1^) (Table 1). Concentrations of the metal showed moderate geographic variability ranging from 295 to 723 μg L^-1^ (RSD: 23%). Considerably higher spatial variability was determined for the second in terms of concentration Mn (mean: 76 μg L^-1^). Its content in the waters of Vistula Lagoon varied from less than 3 to 278 μg L^-1^ (RSD: 103%). Mean concentrations of the remaining trace elements were considerably lower, and did not exceed 10 μg L^-1^: Fe (mean 7.8 μg L^-1^) > Cu (mean: 6.0 μg L^-1^) > Zn (5.4 μg L^-1^) > Ni (mean: 2.3 μg L^-1^) > Pb (mean: 2.1 μg L^-1^) > Se and Cr (mean: 1.7 μg L^-1^) > U (mean: 1.2 μg L^-1^) > Al (mean: 1.1 μg L^-1^). Among them, Pb deserves particular attention. It showed very broad variability from below 0.1 to 25.4 μg L^-1^ (RSD: 303%). Considerable fluctuations also concerned concentrations of Fe (RSD: 83%), Zn, and Al (RSD: 69%). The lowest concentrations, below 1 μg L^-1^, were recorded for Mo (mean: 0.85 μg L^-1^) > As (mean: 0.74 μg L^-1^) > V (mean: 0.38 μg L^-1^) > Co (mean: 0.26 μg L^-1^) > Sb (mean: 0.24 μg L^-1^) > Cd and Ag (mean: 0.05 μg L^-1^) > Tl (mean: 0.01 μg L^-1^). The highest spatial variability in this group of metals was reached by concentrations of Pb (RSD: 100%), Ag (RSD: 80%), and Co (RSD: 46%) (Table 1).

## Discussion

**Fig. 3:**
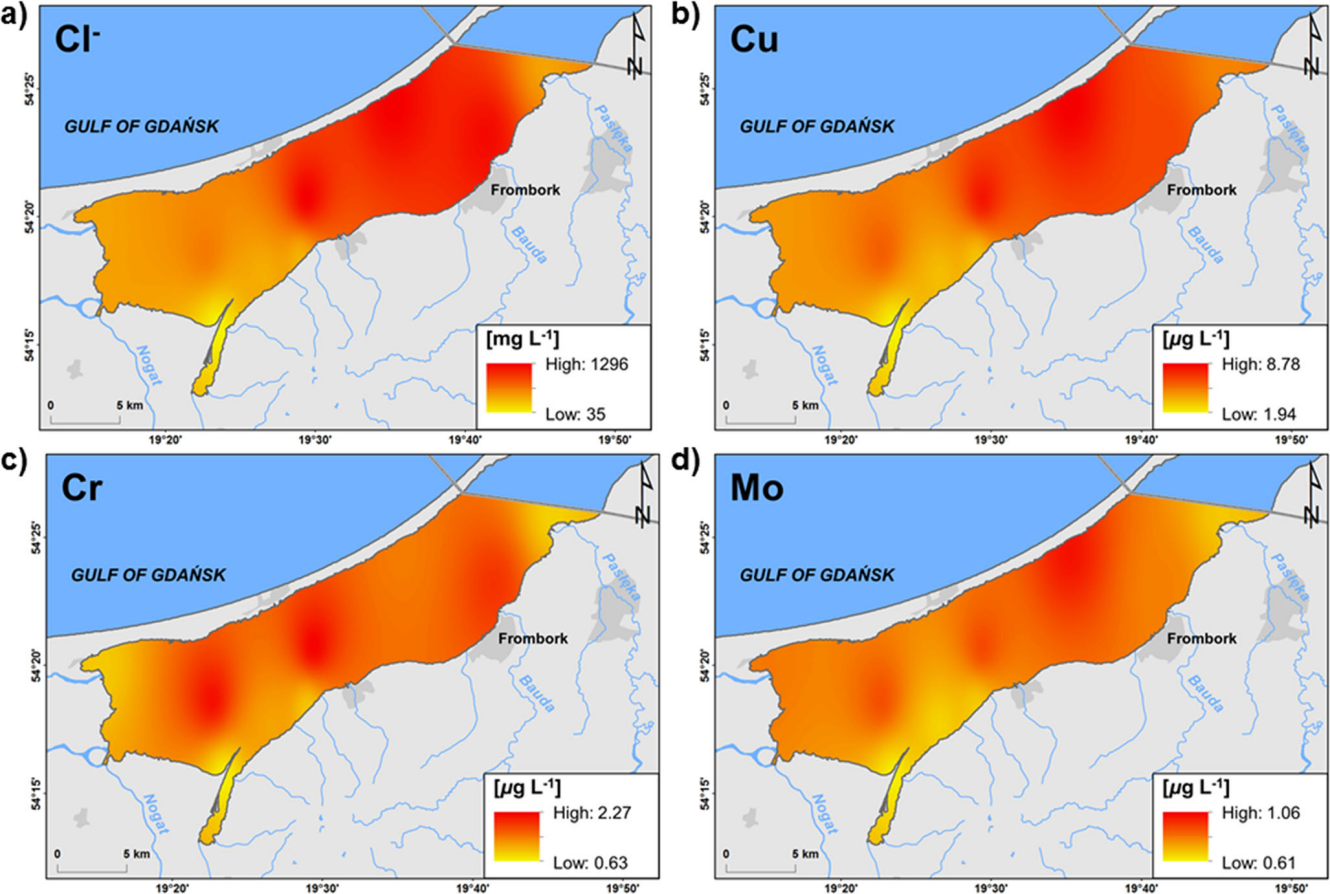
Spatial distribution of ions and metals concentration indicating their inflow with sewater (interpolated from point data). **a** Chlorides (Cl^-^). **b** Copper (Cu). **c** Chromium (Cr). **d** Molybdenum (Mo)

Ice cover on a lagoon prevents wind mixing of water, which contributes to the formation or strengthening of a halocline. In the case of the Vistula Lagoon, despite the presence of ice cover, a horizontal inflow of freshwater from land occured in the south-western part of the lagoon with an inflow of rivers such as Elbląg, Nogat, Szkarpawa, and Wisła Królewiecka, as well as inflow of salty water in the north-eastern part of the estuary through the Strait of Baltiysk (Fig. 1). This is reflected in the spatial distribution of the concentration of chlorides (Fig. 3). A similar distribution was determined for the concentration of the sodium, potassium, magnesium, strontium, and sulphates (Figs. S4 and 6b) as well as soluble: copper, chromium, and molybdenum, suggesting transport of these elements from the Gulf of Gdańsk or from Russian part of the Vistula Lagoon (Fig. 3). The highest concentrations of these metals were determined on stations with the highest salinity (Fig. S2): station 2: Mo 1 μg L and station 3: Cu: 8 μg L^-1^ and Cr: 2 μg L^-1^, confirming their marine origin. The range of impact of river water supplied to the Vistula Lagoon is well illustrated by the Sentinel-1 satellite synthetic aperture radar image showing zones free from ice in river mouths (Fig. 2).

On the deepest stations (3, 5, 9), where water samples were collected from three depths (surface water, middle of the water column, and near-bottom water), the variability of the analysed chemical elements in the vertical profile was determined. In each case, the concentration of marine macroelements increased down the water column and was the highest above the sediment (Fig. S3). The persistence of denser, more saline water near the bottom was confirmed by measurements of salinity and chlorides concentration (Fig. S3). The lowest concentration of chlorides was also analysed in the near-bottom layer on the westernmost station 5 (Fig. 3; Fig. S3). Among these three stations, station 5 is located the nearest to the mouths of relatively large rivers located in the western part of the lagoon (Elblag, Szkarpawa, Nogat, Wisła Królewiecka), resulting in the dilution of the surface water layer with the freshwater (Fig. 1). On the three discussed stations, an increase in salinity in the water column was accompanied by an increase in the concentration of soluble copper and zinc. The highest concentration of both metals was determined in near-bottom water on station 3: Cu: 11 μg L^-1^; Zn: 16 μg L^-1^ (they were also the highest values among all the analysed stations), and the lowest in the vicinity of Frombork, on station 9 (Fig. S3). These metals are important components of organic matter. They can be released back to the water as a result of their degradation. The presence of ice cover and a halocline probably caused several times smaller oxygen concentration on these three deepest stations (Fig. S3). Soluble copper and zinc concentrations above the bottom were the highest on station 3, where O_2_ concentration was the lowest, and water turbidity the highest, suggesting possible desorption of Cu and Zn and organic matter degradation next to the supply of copper from the eastern part of the lagoon (Peng et al. 2009; Zhang et al. 2014).

**Fig. 4:**
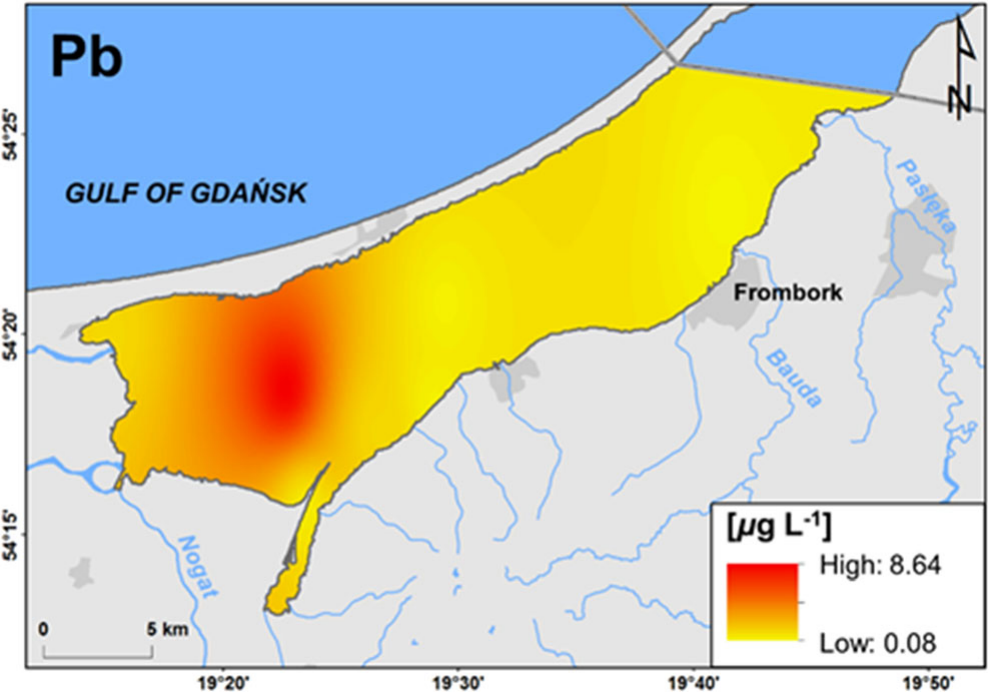
Spatial distribution of lead (Pb) concentration indicating its remobilisation from bottom sediments (interpolated from point data)

**Fig. 5:**
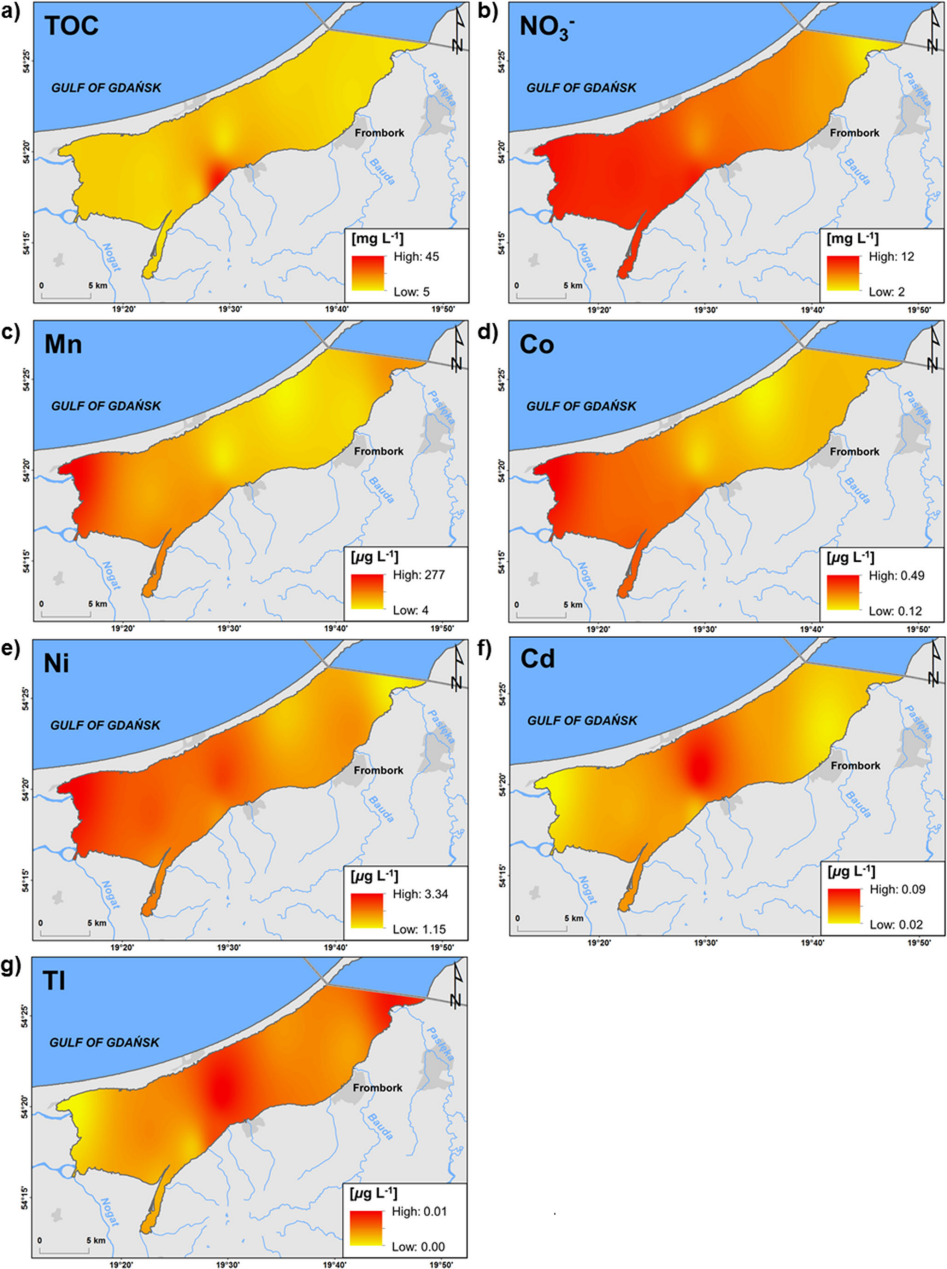
Spatial distribution of ions and elements concentration indicating their inflow from the rivers (interpolated from point data). **a **Total organic carbon (TOC). **b** Nitrates (NO_3_^-^). **c** Manganese (Mn). **d** Cobalt (Co). **e** Nickel (Ni). **f** Cadmium (Cd). **g** Thallium (Tl)

On station 5, located nearest to the mouths of large rivers, where the difference in concentration of marine macroelements between the surface layer and near-bottom water was the highest, 63 times higher soluble lead concentration near the bottom was observed (25 μg L^-1^) in comparison to the surface (0.04 μg L^-1^) (Fig. S3). The station is under the dominant influence of land. Over a year, when the temperature is > 0 °C, metals are introduced here with surface run-off, where due to relatively high depth (2.5 m) as for Vistula Lagoon, they are deposited in sediments. Therefore, Pb can be subject to remobilisation back to near-bottom water, and the presence of a halocline limits its transport and contributes to an increase in its concentration above the sediment. Considering the mean lead concentration in the water column (8 μg L^-1^), it was an area with a concentration several times higher in comparison to the remaining stations (Fig. 4). In the near-bottom water on station 3, 13 times higher soluble manganese concentration was recorded (32 μg L^-1^) than in the surface layer (2.5 μg L^-1^). Mn is a component of sediment; therefore, this case also points to the remobilisation of the metal from sediment. On stations 5 and 9, the situation was the opposite: Mn concentration was nine times higher in the surface layer (193 μg L^-1^ and 63 μg L^-1^, respectively) in comparison to near-bottom water (21 μg L^-1^ and 7 μg L^-1^, respectively) (Fig S3). The analysis of the remaining analysed stations showed an increase in manganese concentration on stations near land in the western and south-western part of the lagoon (Fig. 5). It is most probably related to the leaching of Mn from soil catchment and its transport by rivers under the ice. In this case, the halocline could have caused an increase in the concentration of the metal in the top layer of the lagoon. Similar tendencies were observed in the vicinity of the mouth of Nogat and Szkarpawa in reference to cobalt (0.5 μg L^-1^), nickel (3 μg L^-1^), and nitrates (11 mg L^-1^). Despite their small size, the Grabianka and Olszanka Rivers were an evident source of TOC (41 mg L^-1^) as well as cadmium (0.1 μg L^-1^) and thallium (0.01 μg L^-1^). The Pasłęka River was also a source of the latter (Fig. 5).

**Fig. 6:**
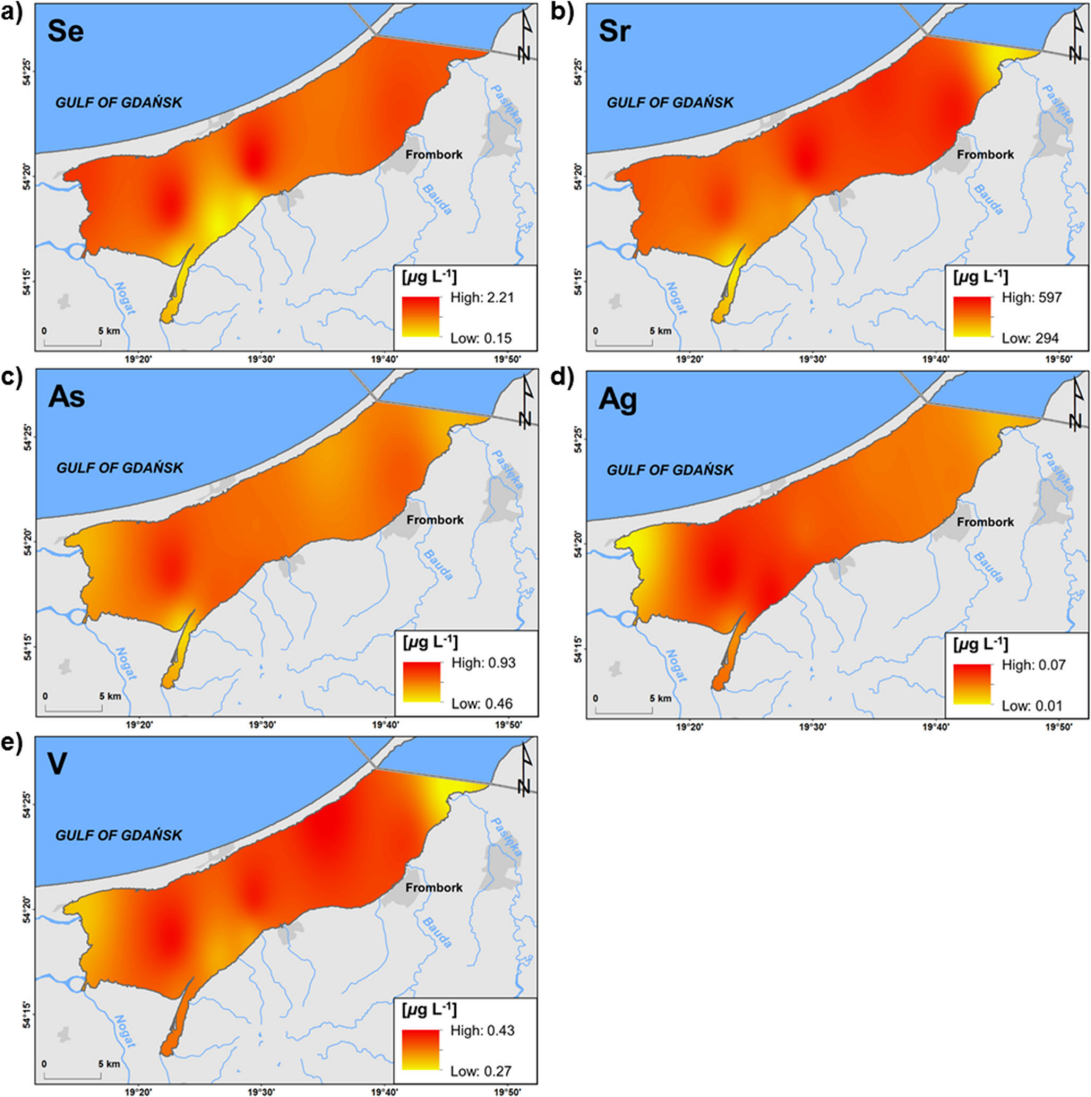
Spatial distribution of elements concentration indicating the "purifying" effect of rivers (interpolated from point data). **a** Selenium (Se). **b** Strontium (Sr) **c** Arsenic (As) **d **Silver (Ag) **e** Vanadium (V)

The Vistula Lagoon is a reservoir of chemical elements leached from the land for decades. The load of pollutants (including untreated industrial wastewaters) was particularly intensive in the second half of the twentieth century. As a consequence, sediments of the lagoon are a potential source of metals to water. On the other hand, during the persistence of the ice cover, the “purifying” effect of rivers was manifested in the western part of the lagoon. Considerably lower concentration of selenium (< 0.0009 μg L^-1^) and strontium (295 μg L^-1^) was recorded in the mouth of the Elbląg River; arsenic (0.5 μg L^-1^) in the mouth of the Elbląg and Nogat Rivers; silver (0.006 μg L^-1^) in the mouth of the Szkarpawa and Nogat Rivers; and vanadium (0.3 μg L^-1^) in the mouth of the Pasłęka River (Fig. 6). This points to the supply of waters from land cleaner in terms of concentration of these chemical elements, and to the importance of rivers in the water balance and matter circulation in estuaries.

Ice cover on the lagoon contributed to the accumulation of chemical substances between the ice and sediment. It was of particular importance in the mouths of rivers transporting chemical elements from land, including nutrients. As a result, in river mouths where the water column was not completly frozen, planktonic organisms could develop. The highest phytoplankton biomass was identified in the vicinity of station 4: 9 mg ww L^-1^ (near the Szkarpawa River) and station 9: 6.8 mg ww L^-1^ (the Bauda River) (Fig. S5). A decrease in iron concentration was also observed (4.2 μg L^-1^ and 4.6 μg L^-1^, respectively), a substance necessary for the development of phytoplankton, as well as uranium (0.939 μg L^-1^ and 0.909 μg L^-1^, respectively) which can be adsorbed by organic matter. In the vicinity of station 9, a decrease in zinc concentration was also analysed (4 μg L^-1^). It also constitutes an important component of the flora (Fig. S4). Dinophyta were the dominant phyla there, and in the western part of the estuary, where the highest phytoplankton biomass was dominated by Cryptophyta (Kornijów et al. 2020), no decrease in Zn concentration was determined. Higher than average phytoplankton biomass was also observed in the vicinity of station 5. It is at a relatively large distance from water supply from land, but it showed the highest (among the analysed stations) water temperature: 1.6 °C (Fig. S2) which could have permitted the development of microorganisms. Higher than average zooplankton biomass was also recorded there in comparison to the remaining stations (Kornijów et al. 2020). The station was also an area with the highest concentration of soluble lead in water (Fig. 4). The Pb level measured in the surface and subsurface water (0.04–0.46 μg L^-1^) was close to the typical Pb concentrations in the aquatic environment that varies from 0.02 to 0.05 μg L^-1^ in marine, and from 0.18 to 1.00 μg L^-1^ in freshwater ecosystems (ILA 2020). However, the Pb concentration in the near-bottom water (25.41 μg L^-1^) was many times higher, suggesting the possible introduction of Pb to the food web. Such a high Pb level in water exceeds the allowable Pb concentrate on in water (7.2 μg L^-1^) set by the European Union (EC 2008) and can cause acute or chronic effects on organisms (Van Sprang et al. 2016; DeForest et al. 2017).

## Summary

In the second half of the twentieth century, the Vistula Lagoon recived a supply of untreated industrial and municipal wastewater. In the early twenty-first century, the situation largely improved — sewage is directed to the treatment plant, although other factors have currently appeared that can affect the level of concentration of metals in the lagoon. In the twentieth century, winters were cold enough for the Vistula Lagoon to be completely frozen even from December to March (Herman 2018). Since the 1990s, warming of the winter season has been observed (Kożuchowski and Degirmendžić 2005; Czernecki and Miętus 2017), and consequently a reduction of the ice period or lack of ice cover (Herman 2018). This affects the circulation of chemical elements in the estuary. As shown by research conducted in 2018, the ice cover efficiently limits wind mixing, changing horizontal transport of elements, and stabilising the halocline, affecting vertical transport. The latter on the one hand strengthens the process of ion remobilisation (e.g. lead), leading to an increase in the concentration of chemical elements in the near-bottom layer. On the other hand, it causes an increase in the concentration over the halocline of chemical substances introduced to the lagoon under ice together with freshwaters. The research also evidenced the cleaning effect of rivers: in the vicinity of river mouths, a decrease was observed in the concentration of chemical elements the supply of which is currently lower input of them than at the end of the twentieth century. The intensive supply of pollutants to the Vistula Lagoon in previous decades is currently also manifested through the input of some elements together with salt waters from the north-eastern part of the lagoon. The remobilisation of chemical elements (including toxic elements) from land and sediments, nowadays, becomes an essential process, when anthropogenic emissions are being reduced.

The described processes result in the accumulation of metals (including toxic ones) in certain areas of the estuary. It may consequently lead to their accumulation by the occurring phyto- and zooplankton (Kornijów et al. 2020) and benthic organisms, and therefore to their introduction to the food web. Previous research described the bioconcentration factor (BCF) of Cd, Pb, Cu, Ni, Zn, and Mn and macroelements Ca, Mg, Na, and K in algae ranged within 3–4 orders of magnitude and was the highest for Zn (amounting to over 500), Pb, and Cd (exceeding 100) (Żbikowski et al. 2006). According to the study by Zalewska et al. (2020) conducted in 2011, these coefficients were even higher reaching 10^4^ for Zn and Cd and 10^3^ for Pb in most of the studied species. The analysis of heavy metals Cu, Zn, Mn, and Fe concentration in benthic organisms carried out in 2001–2003 on the example of polychaete *Marenzelleria virdis* also indicated the uptake of metals from the sediments and surrounding water (Bełdowska and Sokołowski 2018). Elements dissolved in water, bound to suspended matter or sediment particles, are bioaccumulated in the organisms from higher trophic levels, including fish (Kosior et al. 2002; Polak-Juszczak 2009). For example, the Pb bioaccumulation factor (BAF) in perch tissues in 2014 was estimated at 30 (Mazur-Chrzanowska et al. 2016). However, taking into account the concentration of heavy metals in fish caught from the Vistula Lagoon and intended for human consumption, it does not exceed the safety levels. It is of paramount importance as the Vistula Lagoon fulfils a number of essential functions in the scope of both tourism and economy, including fishery (Rychter 2018).

## Supplementary information

ESM 1 (DOCX 2999 kb)
