# Supplementary material for: The impact of sediment, fresh and marine water on the concentration of chemical elements in water of the ice-covered lagoon
Source: Environ Sci Pollut Res Int. 2021 Jun 25;28(43):61189–200. doi: 10.1007/s11356-021-14936-w (PMC8580916; doi:10.1007/s11356-021-14936-w)
Supplement: Supplementary file 1 — (DOCX 2999 kb) [file 11356_2021_14936_MOESM1_ESM.docx]

**SUPPLEMENTARY MATERIAL**

**Table S1** Limit of quantification (LOQ), limit of detection (LOD), precision and accuracy for ICP-MS method

| Chemical element | LOQ  [μg L^-1^] | LOD  [μg L^-1^] | Precision [%] | Accuracy  [%] |
| --- | --- | --- | --- | --- |
| Na | 39.90 | 13.30 | 1.13 | 8.85 |
| K | 28.34 | 9.45 | 0.80 | 3.27 |
| Mg | 35.73 | 11.91 | 1.01 | 6.81 |
| Sr | 0.56 | 0.19 | 1.59 | 1.76 |
| Ca | 47.60 | 15.87 | 1.34 | 4.64 |
| Al | 5.14 | 1.71 | 14.5 | 15.8 |
| Sb | 0.41 | 0.14 | 1.14 | 2.99 |
| As | 0.41 | 0.14 | 1.16 | 4.60 |
| Cr | 0.39 | 0.13 | 1.11 | 3.36 |
| Cu | 0.25 | 0.08 | 0.72 | 2.50 |
| Fe | 31.76 | 10.59 | 0.90 | 0.54 |
| Pb | 1.07 | 0.36 | 3.03 | 4.06 |
| Mn | 0.29 | 0.10 | 0.83 | 3.81 |
| Mo | 0.54 | 0.18 | 1.53 | 6.31 |
| Ni | 0.33 | 0.11 | 0.94 | 3.99 |
| Se | 0.53 | 0.18 | 1.50 | 6.01 |
| Ag | 0.56 | 0.19 | 1.59 | 1.74 |
| U | 0.90 | 0.30 | 2.55 | 4.84 |
| V | 0.57 | 0.19 | 1.60 | 3.45 |
| Zn | 1.34 | 0.45 | 3.78 | 4.19 |
| Cd | 0.33 | 0.11 | 0.93 | 1.13 |
| Co | 0.35 | 0.12 | 1.00 | 2.95 |
| Tl | 0.48 | 0.16 | 1.35 | 2.41 |

**Figure S1** Duration of ice cover in days over sampling points in year 2018 estimated from Sentinel-1 SAR data

**
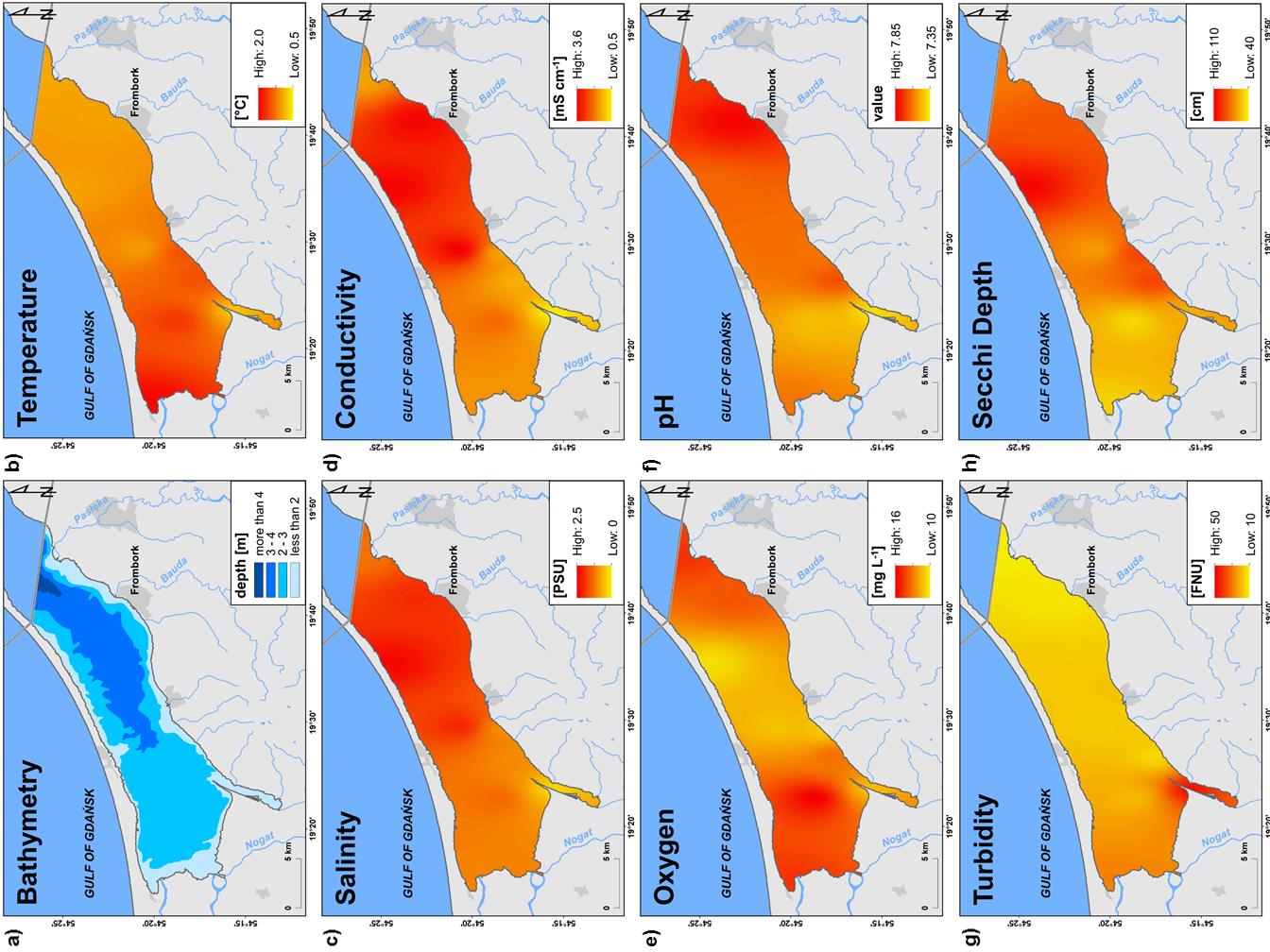
**

**Figure S2** Bathymetry of the Vistula Lagoon and the basic physico-chemical parameters during sampling (interpolated from point data)


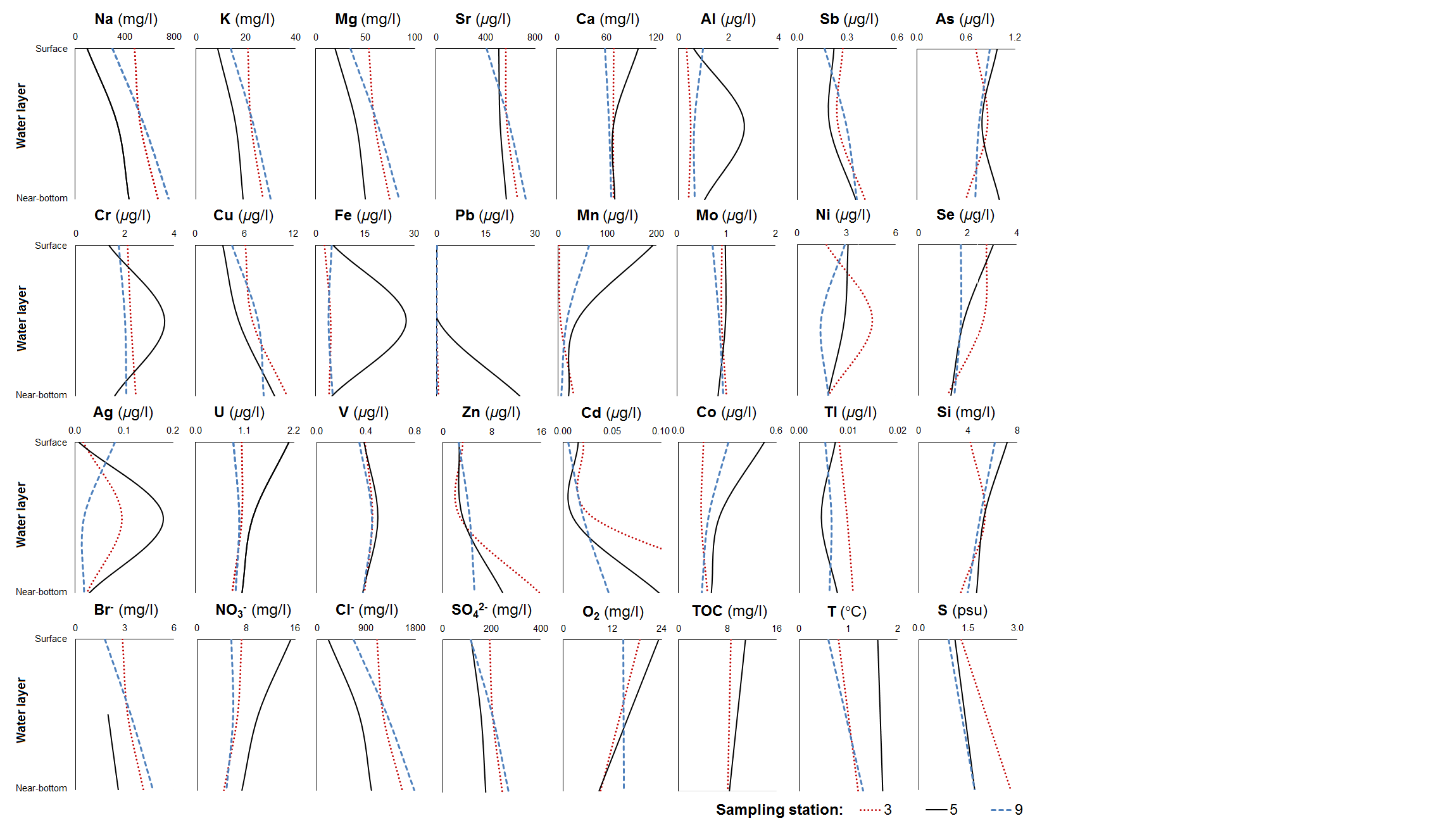


**Figure S3** Concentration of the chemical elements. ions and physico-chemical parameters in the water column at the three deepest sampling stations in the Vistula Lagoon


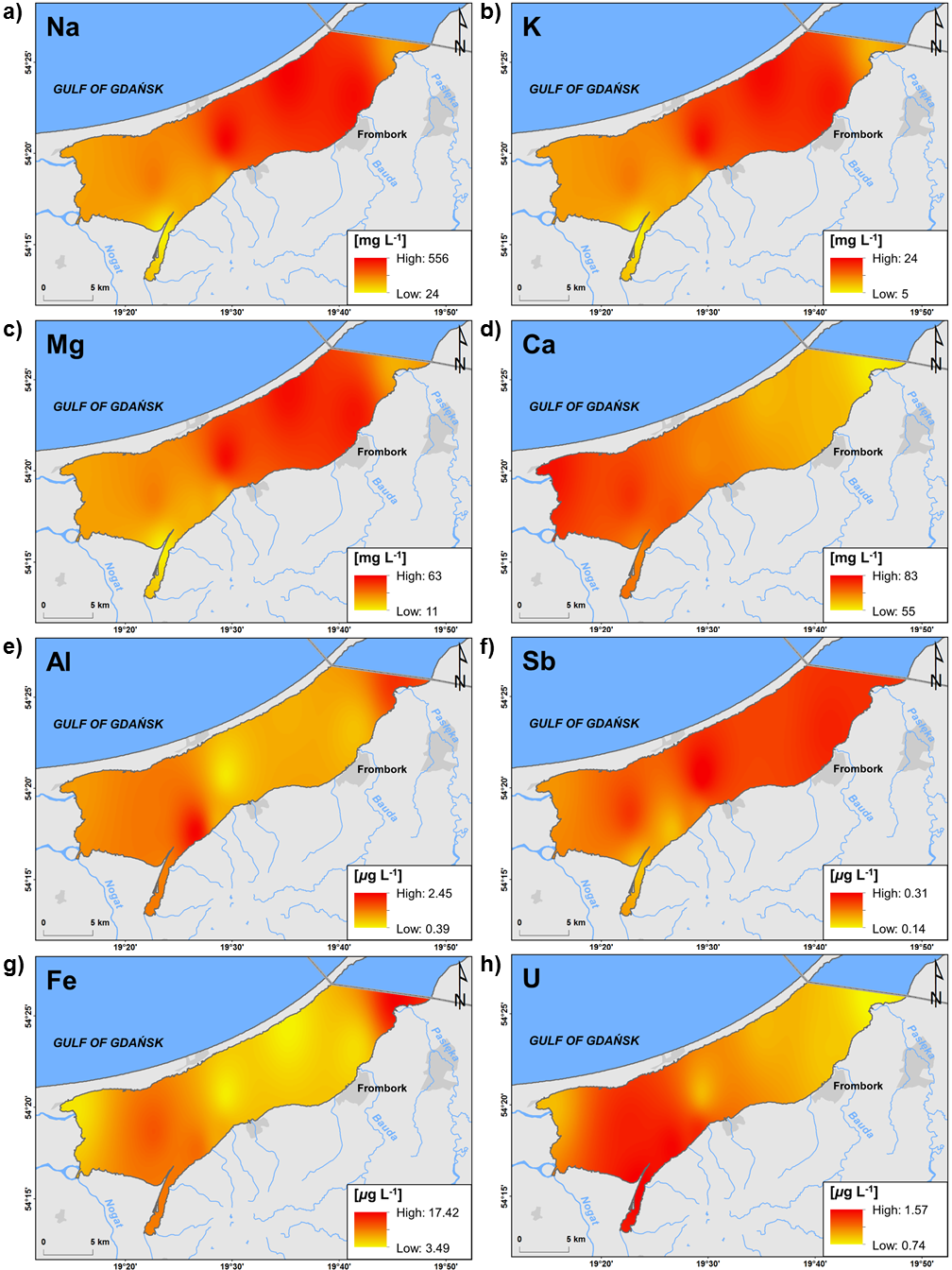


**Figure S4** Concentration of the other analyzed chemical elements and ions in the surface waters of the Vistula Lagoon (interpolated from point data)


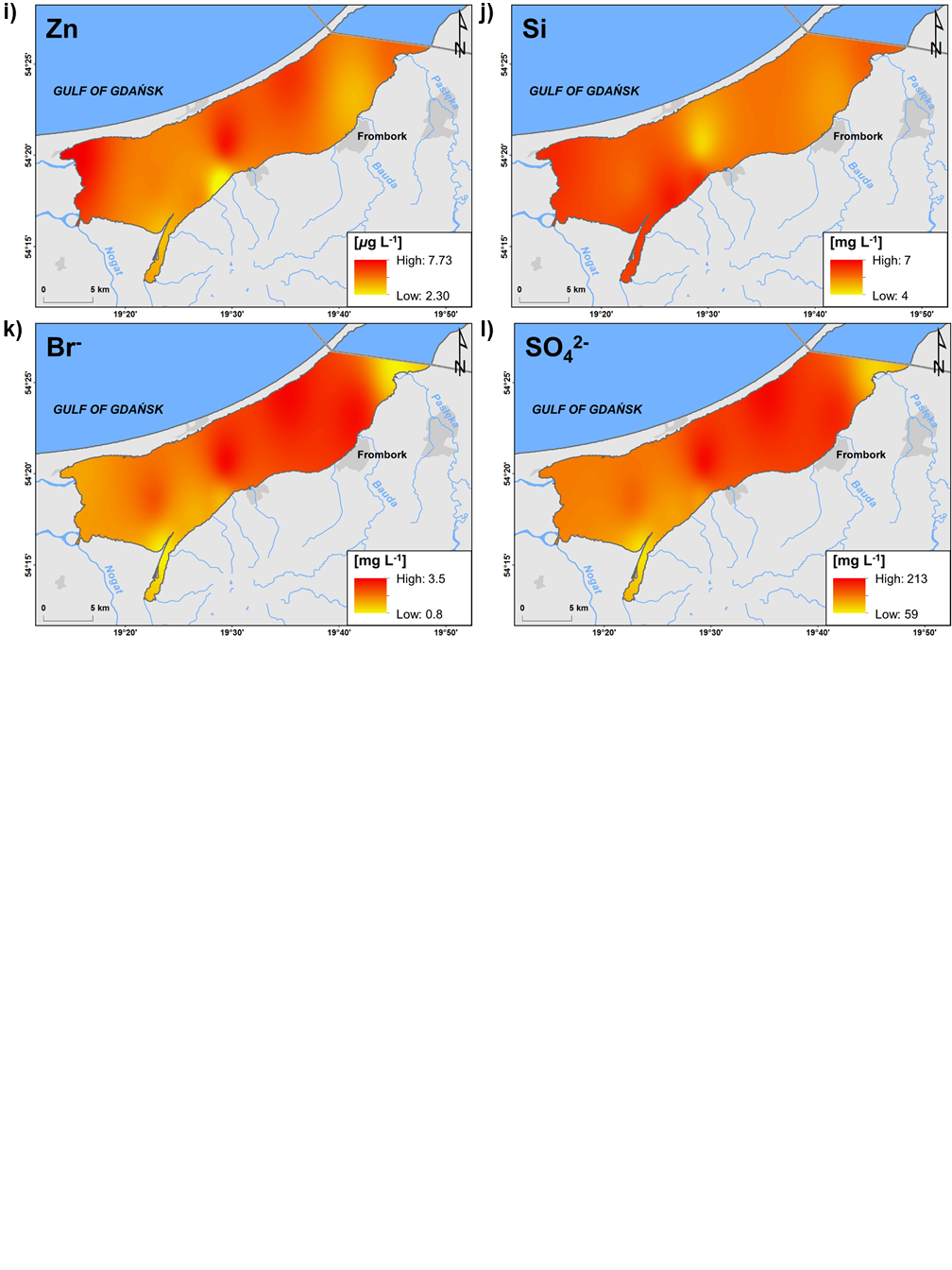


**Figure S4** Continued


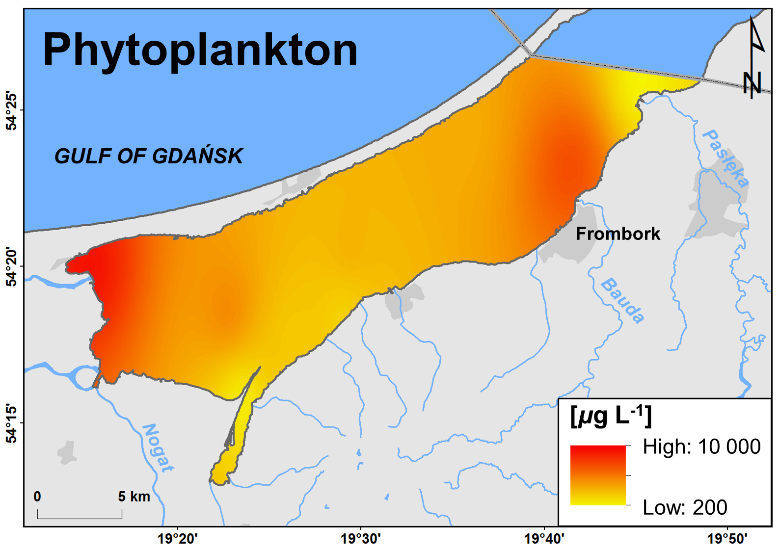


**Figure S5** Concentration of phytoplankton in the surface waters of the Vistula Lagoon (interpolated from point data)
